# Risk factors for death of follicular thyroid carcinoma: a systematic review and meta-analysis

**DOI:** 10.1007/s12020-023-03466-9

**Published:** 2023-10-07

**Authors:** Ting Zhang, Liang He, Zhihong Wang, Wenwu Dong, Wei Sun, Ping Zhang, Hao Zhang

**Affiliations:** Department of Thyroid Surgery, The First Hospital of China Medical University, Shenyang, Liaoning Province 110001 China

**Keywords:** Follicular thyroid carcinoma, Death, Risk factors, Cervical lymph node metastasis, Non-radical resection.

## Abstract

**Background:**

There are conflicting reports on the factors that increase the likelihood of patients dying from follicular thyroid carcinoma (FTC). Therefore, it is critical to identify risk factors of patients with FTC. This study aimed to identify the factors that increase the risk of death of patients with FTC and help clinicians make better treatment and follow-up decisions.

**Methods:**

A systematic literature review was conducted in PubMed and Web of Science databases for relevant studies published before January 31, 2023. Their reference lists were also analyzed. Two reviewers extracted data and evaluated the quality of eligible studies independently. Studies on patients who had open thyroidectomy procedures with or without neck dissection were included in this review. The RevMan 5.3 software was used to analyze the data.

**Results:**

This meta-analysis included thirteen studies with a total of 2075 patients. The following variables were associated with an increased risk of death in FTC patients: age > 45 years, male, tumor diameter > 4 cm, multifocality, extrathyroidal extension (ETE), widely invasive (WI), cervical lymph node metastasis (CLNM), distant metastases (DM) and non-radical resection tumor. Lobectomy and no radioactive iodine (RAI) treatment was not associated with the death of FTC patients.

**Conclusion:**

Clinicians should pay closer attention to the following significant risk factors associated with the death of FTC patients: age (> 45), male, multifocality, tumor diameter > 4 cm, ETE, WI, non-radical resection tumor, CLNM, and DM. Individualized initial treatment and close follow-up are needed FTC patients who have these risk factors.

## Introduction

Follicular thyroid carcinoma (FTC) accounts for approximately 10% of all thyroid cancers [1]. FTC and papillary thyroid carcinoma (PTC) have been classified as differentiated thyroid cancers because they emerge from the follicular thyroid cell lineage [2]. On the other hand, there is a marked distinction between the biological characteristics and clinical manifestations of FTC and PTC [3]. Compared to PTC, FTC is considered a more aggressive disease with a poor prognosis. Because of the propensity for capsular and vascular invasion, distant metastases via hematogenous dissemination are more common in FTC [4].

FTC patients typically have a good prognosis, but they may experience local recurrence, distant metastasis, or even death during the follow-up period [5]. According to recent studies, the mortality rate of FTC was 10–30% [6, 7]. Therefore, the most important question among endocrine treating physicians is whether treatments, such as completion thyroidectomy and radioactive iodine (RAI) remnant ablation, should be performed after FTC diagnosis, and what FTC patients can benefit from these treatments in terms of prognosis [8]. The decision to continue treatment should be based on prognostic indicators and risk factors for death [9], even though the pathological tumor node metastasis classification has been established in patients with well-differentiated thyroid carcinoma. Indeed, due to the low number of FTC cases, FTC and PTC are frequently analyzed together in many published studies [10]. However, this joint analysis is inaccurate because the biological behaviors of PTC and FTC are differ [11, 12]. Furthermore, prognostic indicators are not identified in FTC and have not yet been validated for FTC [13].

In FTC patients, various prognostic factors were documented, including age at diagnosis, gender, tumor size, extrathyroidal extension (ETE), and the presence of distant metastasis [14]. Furthermore, it has been demonstrated that widely invasive carcinoma has worse outcomes than minimally invasive carcinoma [15]. Despite this, there are numerous contradictory reports on the risk factors for FTC death [16–18]. Therefore, a meta-analysis was conducted to investigate the risk factors for death in FTC patients and aid clinical decision-making for appropriate treatment and follow-up.

## Materials and methods

The Preferred Reporting Items for Systematic Reviews and Meta-analysis (PRISMA) guidelines were used to conduct this meta-analysis [19].

### Search strategy

A systematic literature search was conducted in PubMed and Web of Science databases for relevant studies published before January 31, 2023. The keywords included thyroid cancer OR thyroid carcinoma, follicular OR FTC, risk factors, death OR mortality, outcome, survival, prognosis OR prognostic factors. Two authors carried out the selection process independently (Zhang T and Dong WW). All disagreements were resolved by the two authors through discussions and consensus, or referred to a third author.

### Selection criteria

The meta-analysis included prospective or retrospective studies published in English that included primary FTC patients who underwent thyroid and lymphadenectomy surgery. Participants in the included studies were diagnosed using intraoperative or postoperative pathology. Furthermore, the studies included demographic and clinical data for thyroidectomy patients that could be extracted. Review articles, conference abstracts, editorials, letters, and single case reports, on the other hand, were excluded. Duplicate studies were also excluded, as were those without reported outcomes.

#### Data extraction and quality assessment

**Fig. 1:**
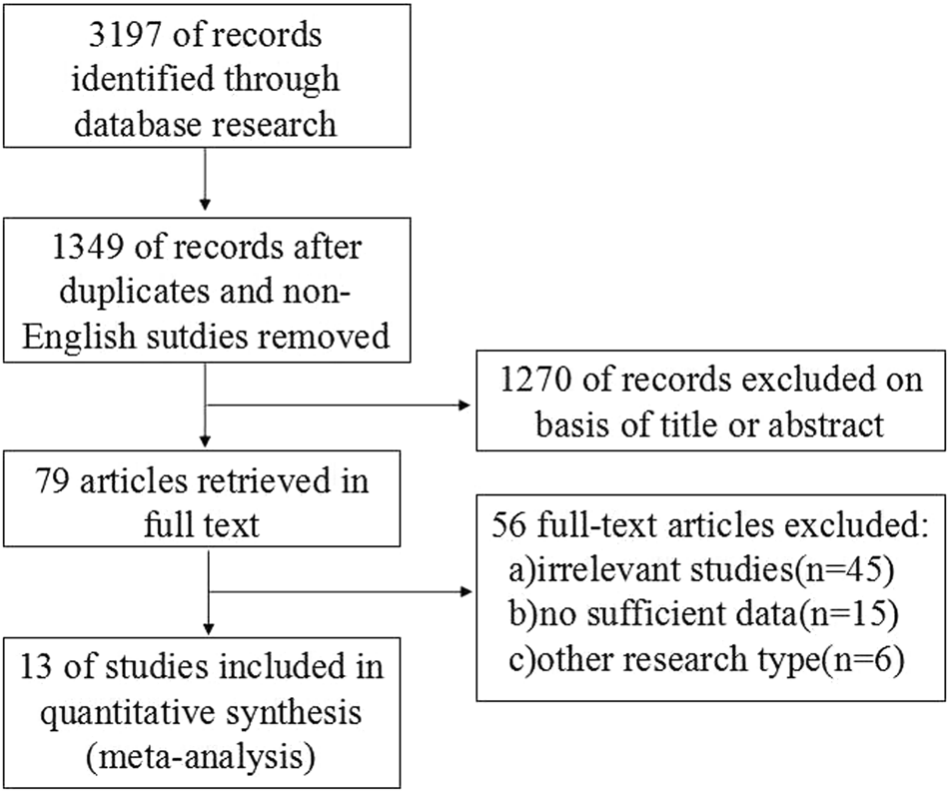
Flowchart of study selection

The two investigators independently extracted relevant data from the articles in a standardized format. The data included the first author’s name, year of publication, country of origin, research design, number of cases, potential risk factors, and other corresponding data (Fig. 1). The potential risk factors included demographic variables (age, gender), tumor specific variables (tumor diameter, multifocality, ETE, pathologic subtype (widely invasive (WI), or minimally invasive (MI))), disease extent (cervical lymph node metastasis (CLNM), distant metastasis (DM)), and treatment variables (operation type, margin status, radioactive iodine (RAI)). The Newcastle-Ottawa quality assessment scale was applied to evaluate the quality of studies [20, 21].

### Data analysis

The data were analyzed using Review Manager version 5.3 (Cochrane Collaborative, Oxford, United Kingdom), and the results were presented as mean difference IV (MD) or odds ratios (ORs) with a 95% confidence interval (CI). A *P*-value < 0.05 indicated that the observed difference was statistically significant. The Q test and I2 statistics were used to assess data heterogeneity, while Cochran’s Q statistic was used to estimate heterogeneity among studies [19]. A fixed-effects model was used when *p* > 0.10 and I2 < 50%; otherwise, a random-effects model was used. Begg’s plot was used to assess the potential of publication bias.

## Results

The search strategy yielded 3197 potentially relevant studies for meta-analysis. Figure 1 displays a flowchart of the studies that were retrieved and excluded. After excluding studies that did not meet the inclusion criteria, 13 studies [6, 7, 22–32] with 2075 patients were chosen for analysis. This meta-analysis literature search of our study included Hurthle Cell Carcinoma (HCC), because HCC belong to FTC before the 5th edition (2022) of the WHO Classification of Endocrine and Neuroendocrine Tumors. In this study, 91 patients with a diagnosis of HCC were included. The mean follow-up was 3.9–14.4 years. Carcinoma specifific mortality was calculated as the duration from the point of diagnosis to the date of death from FTC. Furthermore, the estimated cumulative incidences of FTC death at 5 and 10 years were 3.5–47.7% and 2.55–34.5%, respectively. The incidence of death in all studies was represented in Table 1.

**Table 1:** Characteristics of Eligible Studies

Author	Year	Country	Study design	Case number		Follow-up period (years)	FTC death		Quality score
				Death	All		5 years	10 years	
Lin, JD	1999	China	retrospective analysis	25	69	7 (0–18)	47.70%	—	7
Chow, SM	2002	China	retrospective analysis	37	215	10.8 (0–20)	31.20%	—	9
Passler, C	2004	Austria	retrospective analysis	51	168	10.8 (0–20)	30.50%	21.00%	8
Lo, CY	2005	China	retrospective analysis	17	156	14.4 (0.1–38.6)	6.00%	12.00%	8
Pulcrano, M	2007	France	retrospective analysis	12	40	3.9 (0–19.7)	30.00% (2.9years)^a^	—	6
Asari, R	2009	Austria	retrospective analysis	45	207	9.7 (1–34)	18.80%	22.50%	9
de Melo TG	2014	Brazil	retrospective analysis	12	89	9.4 (1–36.6)	13.48%	—	7
Kim, HJ	2014	Korea	retrospective analysis	16	204	3.7 (2.3–8.8)	6.00%	15.00%	9
Stenson, G	2016	Sweden	retrospective analysis	5	58	11.7 (1.8–25.7)	3.50%	34.50%	7
Lee, YM	2016	Korea	retrospective analysis	4	166	8.6 (1.1–20.3)	4.20%	—	8
HUANG Ji-yuan	2016	China	retrospective analysis	3	21	0.25–10	—	24.60%	6
Su, DH	2018	China	retrospective analysis	30	204	8.3 (1–24.4)	10.60%	16.50%	9
Yamazaki, Haruhiko	2020	Japan	retrospective analysis	10	478	7.7	—	2.55%	9

^a^End point of follow-up

### Age

**Fig. 2:**
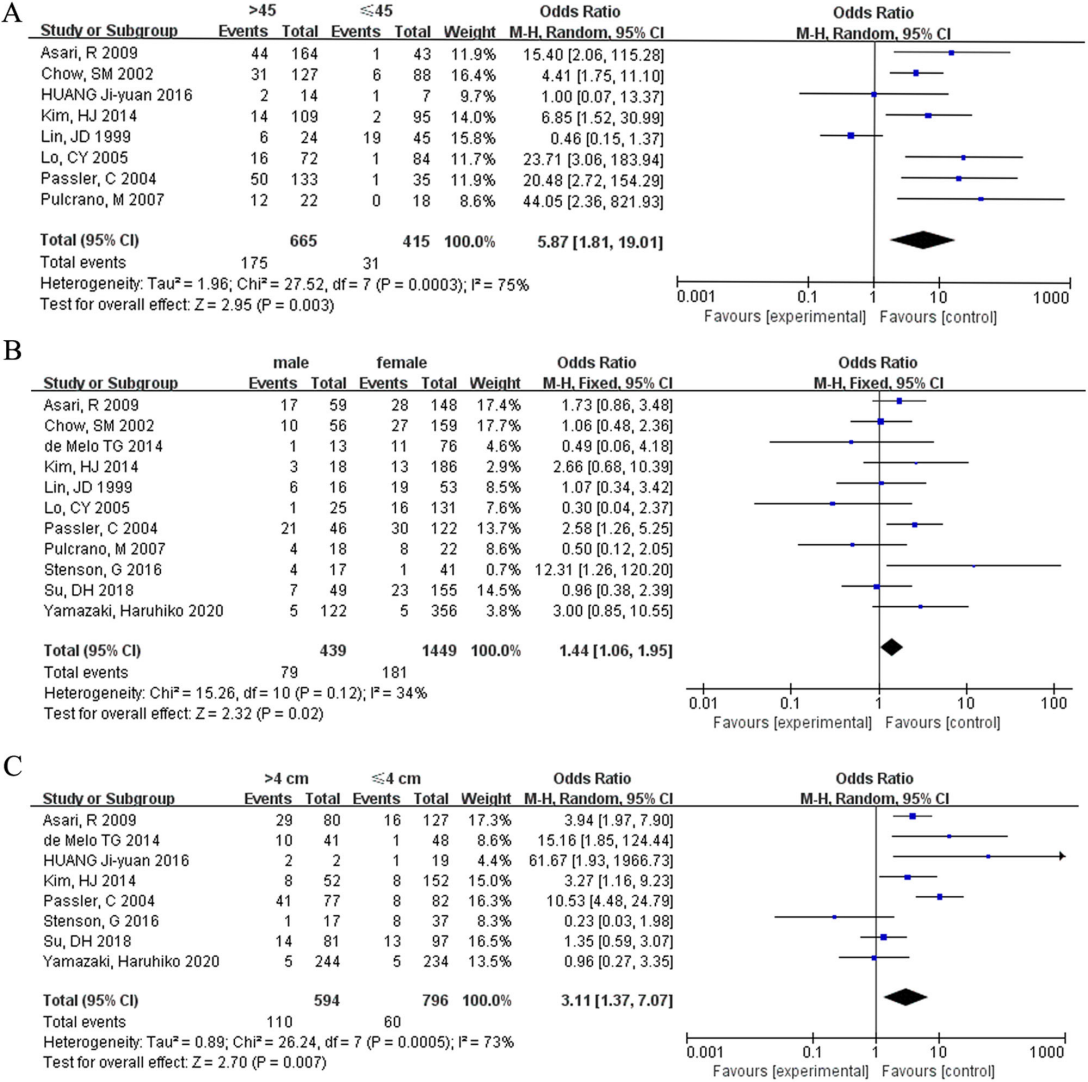
Meta-analysis results for the risk factors of FTC. (**A**) Age; (**B**) gender; (**C**) tumor size

This study included eight studies that looked at age difference in FTC patients aged ≤ 45 and > 45 years. The findings show that age ≤ 45 years was associated with an increased risk of death in FTC patients (OR = 0.17, 95% CI = 0.05–0.55, *p* = 0.003) (Fig. 2A). Many record data of published articles before 2018, and according to the 6th or 7th edition of the AJCC/TNM staging system, the age boundary is 45 years old. A small number of literature reports that the age boundary is 55 or 60 years old, but due to the number of such literature being less than 3, it cannot be included in the RevMan 5.3 software.

### Gender

Eleven studies were included in analyzing the risk factor in FTC patients based on gender (male and female). Male FTC patients died at a significantly higher rate (OR = 0.70, 95% CI = 0.51–0.94, *p* = 0.02) (Fig. 2B).

### Tumor diameter

This analysis included eight studies. Tumor diameter greater than 4 cm was associated with the incidence of death in FTC patients (OR = 0.32, 95% CI = −0.14–0.73, *p* = 0.007) (Fig. 2C).

### Multifocality

**Fig. 3:**
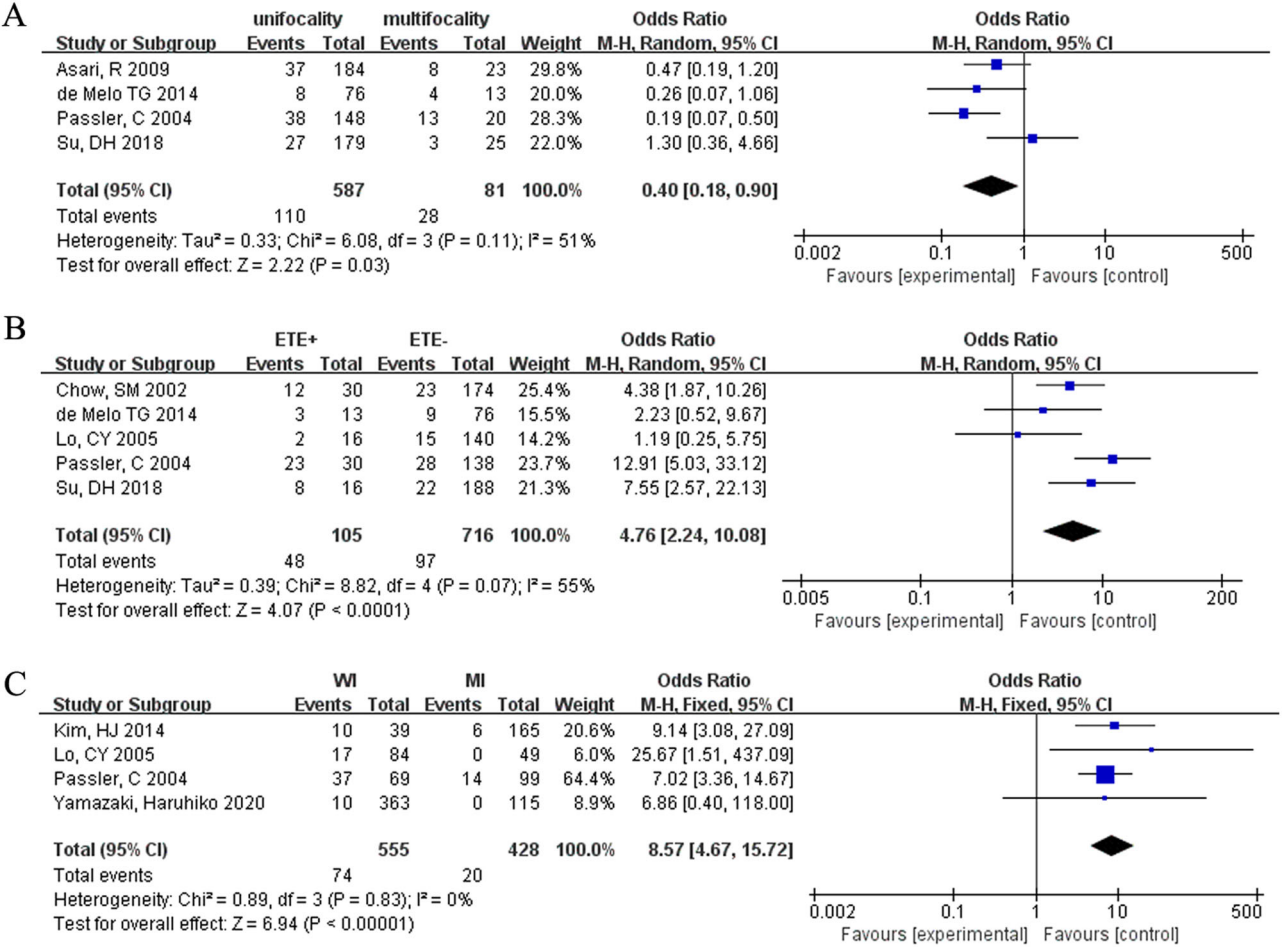
Meta-analysis results for the risk factors of FTC. (**A**) Multifocality; (**B**) ETE; (**C**) histologic subtype

The analysis of tumor multifocality included four studies. In FTC patients, there was a positive correlation between the number of foci and incidence of death (OR = 0.40, 95% CI = 0.18–0.90, *p* < 0.03) (Fig. 3A).

### Incidence of ETE

Five studies were included in this analysis. ETE significantly increased the risk of death in FTC patients (OR = 4.76, 95 % CI = 2.24–10.08, *p* < 0.00001) (Fig. 3B).

### Pathologic subtype

Four studies were investigated. Widely invasive FTC patients had an 8.57-fold increased risk of death (OR = 8.57, 95% CI = 4.67–15.77, *p* < 0.00001) (Fig. 3C).

### Surgical type

**Fig. 4:**
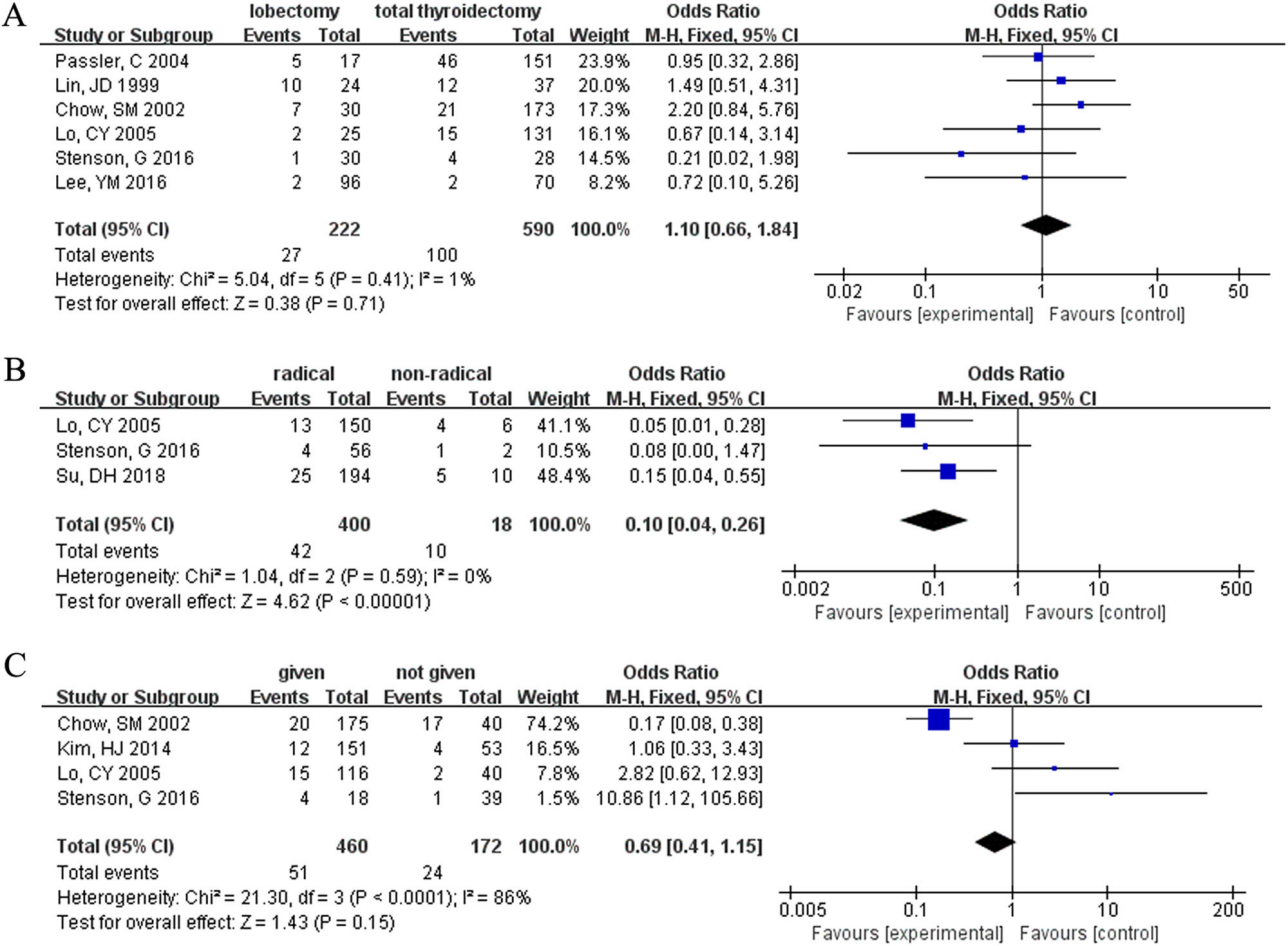
Meta-analysis results for the risk factors of FTC. (**A**) Operation type; (**B**) surgical margin; (**C**) radioactive iodine

The analysis of risk factors for FTC patients based on surgical type included six studies. However, in FTC patients, neither lobectomy nor total thyroidectomy was associated with death (OR = 1.10, 95% CI = 0.66–1.84, *p* = 0.71) (Fig. 4A).

### Surgical margin (microscopical)

This analysis included three studies. In FTC, tumor non-radical resection was associated with a higher risk factor for death than radical resection (OR = 0.10, 95% CI = 0.04–0.26, *p* < 0.00001) (Fig. 4B).

### RAI

A total of four studies were included in our database, and the death of FTC patients was not associated with the administration of RAI (OR = 0.69, 95% CI = 0.41–1.15, *p* = 0.15) (Fig. 4C).

### CLNM

**Fig. 5:**
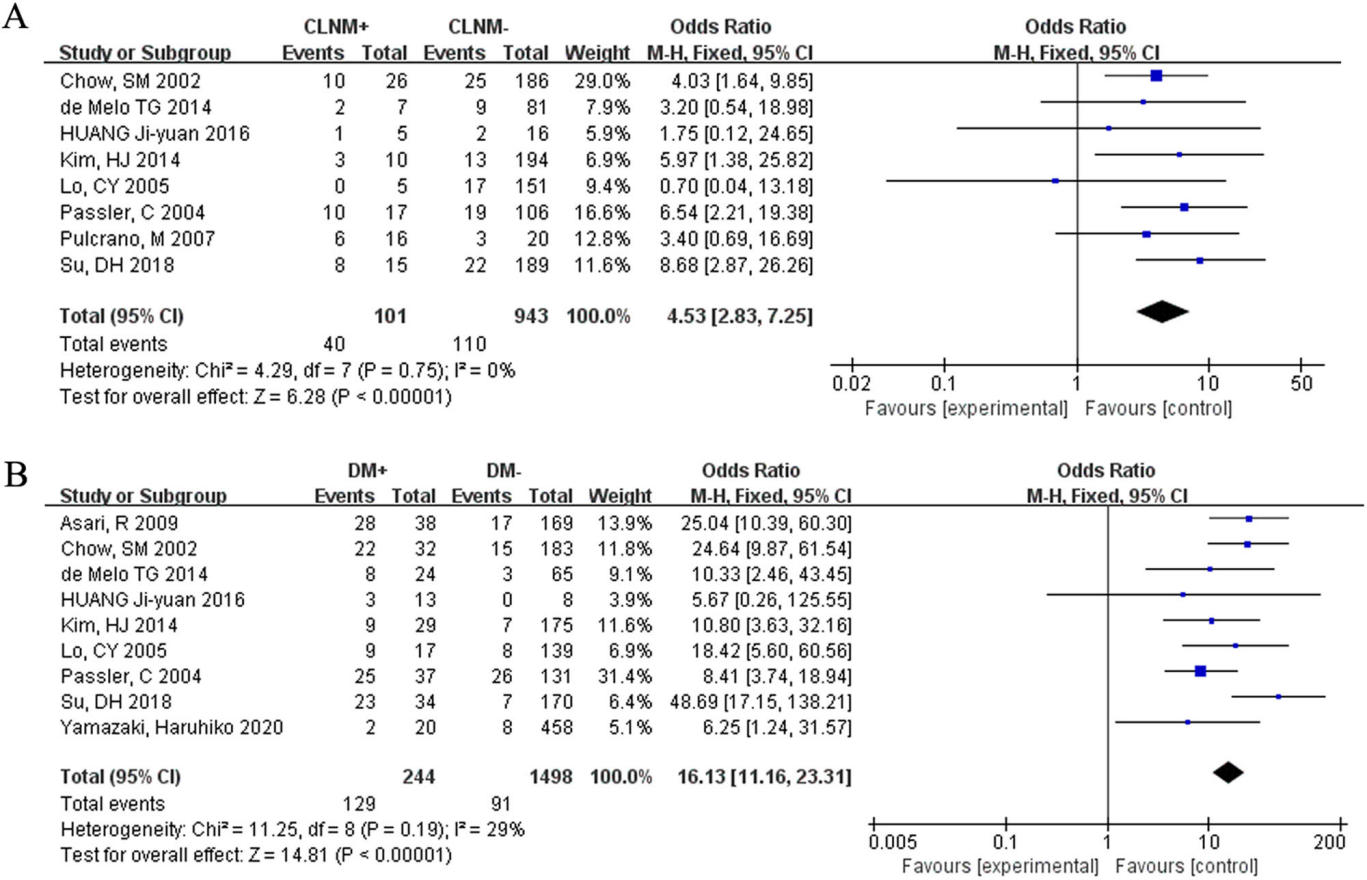
Meta-analysis results for the risk factors of FTC. (**A**) CLNM; (**B**) distant metastasis

The influence of CLNM on death in FTC patients was assessed in six studies. CLNM was linked to a high rate of death (OR = 4.53, 95% CI = 2.83–7.25, *p* < 0.00001) (Fig. 5A).

### DM

There were nine studies included in this analysis. In FCT patients, DM was correlated with a high risk of death (OR = 16.13, 95% CI = 11.16–23.31, *p* < 0.00001) (Fig. 5B).

## Discussion

Although FTC is generally thought have a good prognosis, some studies have described fatal FTC cases [33–35]. Furthermore, several studies have identified some clinical indicators as poor prognostic factors of FTC for long-term patient survival, which remains debatable, such as age, gender, tumor size, ETE, and so on [36–38]. Few studies evaluated these characteristics with the recommended surgical procedure. Thus, the risk factors of death in patients with FTC should be carefully considered and evaluated, particularly in patients undergoing preoperative evaluation. Predictive risk factors for FTC death are helpful for clinicians in assessing the clinicopathological status of FTC and informing the clinical decision-making of treating physicians [8, 39]. The current study is the first meta-analysis to investigate the risk factors for death of FTC patients, and the findings will assist with evidence-based decisions.

Several studies have revealed the risk factors for FTC death. M C Coburn et al. [40] found that older age at diagnosis was strongly associated with increased mortality. The 10-year survival rate for the older age group was 48%, while the younger age group was 92%. Jukkola A et al. [41] suggested that males had a higher death rate than females.

Similarly, in a study, Xuan V et al. [42] discovered that the tumor diameter of the deceased cases was significantly larger than that of the survivors. Most studies [24, 43] found that multifocality is an independent risk factor for death, with FTC mortality in multifocality being significantly higher than in unifocal cancers. FTC is classified into two major categories in the third edition of the WHO classification based on their degree of invasiveness. WI FTC shows widespread infiltration into adjacent thyroid tissues and blood vessels, whereas MI FTC demonstrates limited capsular and vascular invasion. Invasiveness is not readily visible with MI FTC and can only be determined under a microscope. WI FTC is thought to have a worse prognosis than MI [3]. A retrospective study of 318 FTC patients found that FTC-related ETE (10.37% of cases) had a significantly higher mortality rate than non-ETE [44]. Similarly, univariate and multivariate analysis revealed that WI FTC was significantly associated with death; FTC mortality was 20% (5/25) in the WI group and 0% (0/48) in the MI group [45].

The link between CLNM and death is still debatable. According to some studies, CLNM is not an independent risk factor for death in FTC [46, 47]. Other studies, however, report that CLNM was considered a risk factor [5, 38]. For example, Witte J et al. [48] proposed that FTC be considered when selecting patients for prophylactic central neck lymph node dissection. Ito Y and Slook O et al. conducted a multivariate analysis, and their findings showed that CLNM is an independent risk factor for death in FTC [13, 49]. FTC tends to invade blood vessels, leading to hematogenous dissemination, which increases the likelihood that it will metastasize to distant organs rather than to regional lymph nodes. Patients with FTC who have distant metastases at the time of diagnosis have a poor prognosis [16]. Despite the general belief that FTC has a good prognosis, Su DH et al. reported that most FTC patients have distant metastases [6]. A recent multicenter study with a large cohort demonstrated that distant metastases have an independent risk prognostic value in FTC clinical outcomes [50].

According to KC Loh et al. [51], lobectomy was strongly associated with PTC recurrence and death. According to Rao RS et al. [52], patients who undergo total thyroidectomy have an excellent prognosis. During postoperative follow-up, Kim HJ [29] confirmed that the mortality of FTC was not different between lobectomy and thyroidectomy. Most researchers believed that tumor non-radical resection was significantly related to poor survival of FTC patients [25, 53]. Would highlight that margin positivity may reflect either a very extensive tumor or poor surgical technique that may be the independent driver of poor outcome. Previous research has produced conflicting results regarding whether the death of FTC differs when RAI is administered or not. Jen-Der Lin et al. confirmed that total thyroidectomy with RAI therapy for FTC patients was thought to be unnecessary [54]. Furthermore, Aziz A et al. reported that RAI was associated with FTC patient survival [55]. On the other hand, Hay ID believed that not all FTC patients were inappropriate for RAI [56].

Our forest map analysis revealed that age > 45 years, male, multifocality, tumor diameter > 4 cm, ETE, WI, CLNM, DM and non-radical resection of tumor were risk factors for FTC death. The reason could be that these risk factors are linked to tumor aggressiveness and play a significant role in FTC development. However, lobectomy and failure to receive RAI were not risk factors for FTC death. This finding was contradicted to the published results and that could be attributed to difference in the patient selection criteria and study designs. In this study, there is no evidence that the treatment intensity variables are controlled for the other variables. RAI, for instance, would be expected to be applied to tumors with higher demographic or tumor specific risk. Therefore, an alternative explanation for the findings is that RAI abrogated the poorer outcome for these tumors such that the mortality is now similar to those not treated with RAI. The presence of capsular and vascular invasion of FTC cannot be determined by using preoperative fine-needle aspiration cytology and frozen section pathology because the diagnostic criteria are based on postoperative histological sections [57]. Therefore, even with biomarkers and specific stains, early and accurate diagnosis of FTC is difficult because follicular adenoma and FTC are difficult to be distinguished based on cell morphology [58].

According to our findings, whether a lobectomy or a total thyroidectomy is performed is unimportant. However, it is necessary to ensure complete radical resection. This finding is valuable for treating physicians when FTC is suspected during operation. It is important to note that, complete radical resection, including CLND, should be performed avoiding recurrent laryngeal nerve injury and hypoparathyroidism. Moreover, clinicians should use more individualized initial treatment and closer follow-up for FTC with patients age > 45, male, multifocality, tumor diameter > 4 cm, ETE, WI, CLNM, DM and non-radical resection tumor. Individualized FTC treatment and follow-up could be achieved by combining these clinicopathological risk factors with other imaging techniques.

This study has several limitations. First, the number of studies included was limited due to the lack of raw data from some articles. Second, this meta-analysis included several extensive studies, which may have introduced bias in the overall study results. Third, most patients in the included studies were Asian, which could lead to bias if the findings are applied to all races. This study excluded a randomized controlled trial. Fourth, differences in study populations and objectives among the included studies may have resulted in selective bias. The number of patients with or without CLNM was counted regardless of metastatic site, ipsilateral or bilateral, and central or lateral cervical nodes. Fifth, The cumulative incidence range for 5 years is greater than for 10 years, this is because not every study reports both cumulative incidence outcomes, and there may be selective bias that be due to different case selection criteria for the included studies.

## Conclusions

The following significant risk factors for patients dying from FTC were identified in this meta-analysis: age > 45 years, male, multifocality, tumor diameter > 4 cm, ETE, WI, non-radical resection tumor, CLNM, and distant metastases. There was no correlation between lobectomy, RAI treatment and the death of patients with FTC. This finding will help individual management of patients with these risk factors.

### Supplementary information

supplementary Fig

## Data Availability

The datasets used or analyzed during the current study are available from the corresponding author on reasonable request.
