# Supplementary figures and images for: Risk factors for death of follicular thyroid carcinoma: a systematic review and meta-analysis
Source: Endocrine. 2023 Oct 7;82(3):457–66. doi: 10.1007/s12020-023-03466-9 (PMC10618390; doi:10.1007/s12020-023-03466-9)

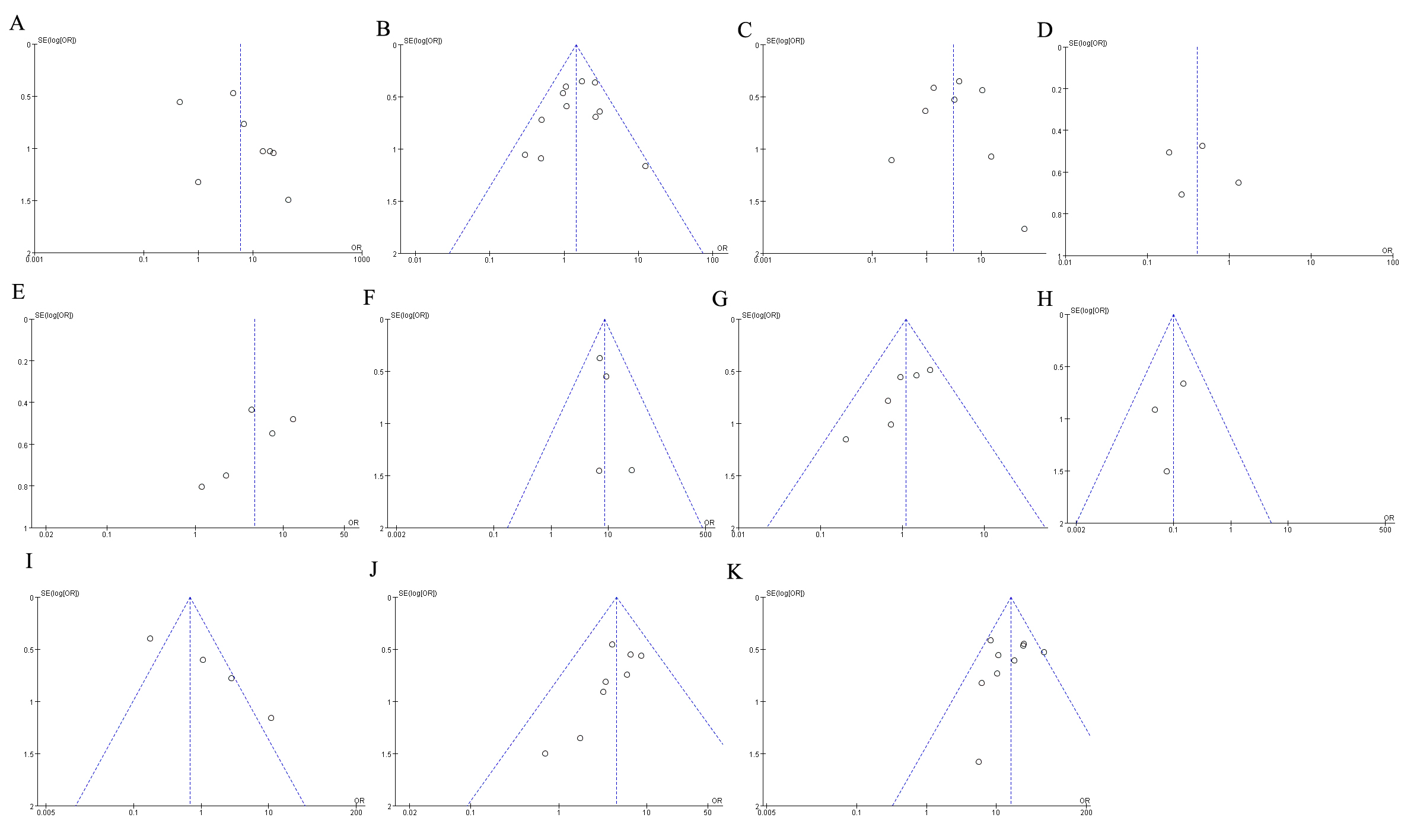

Supplement: Supplementary file 1 — supplementary Fig [file 12020_2023_3466_MOESM1_ESM.jpg]
